# Weight cycling and cardiovascular outcome in women with suspected ischemia: A report from the NHLBI-sponsored WISE Study

**DOI:** 10.1371/journal.pone.0207223

**Published:** 2018-12-03

**Authors:** C. Noel Bairey Merz, Marian B. Olson, Sheryl F. Kelsey, Vera Bittner, Steven E. Reis, Nathaniel Reichek, Eileen Handberg

**Affiliations:** 1 Barbra Streisand Women’s Heart Center, Cedars-Sinai Heart Institute, Cedars-Sinai Medical Center, Los Angeles, California, United States of America; 2 Department of Epidemiology, Graduate School of Public Health, University of Pittsburgh, Pittsburgh, Pennsylvania, United States of America; 3 Division of Cardiology, Department of Medicine, University of Alabama at Birmingham, Birmingham, Alabama, United States of America; 4 Department of Medicine, University of Pittsburgh, Pittsburgh, Pennsylvania, United States of America; 5 St. Francis Hospital, Roslyn, New York, United States of America; 6 Division of Cardiovascular Medicine, University of Florida, Gainesville, Florida, United States of America; University of Bologna, ITALY

## Abstract

**Background:**

We previously reported in a cross-sectional analysis an adverse relationship between weight cycling and HDL-cholesterol but not angiographic obstructive coronary artery disease (CAD) among women undergoing coronary angiography for suspected ischemia in the NHLBI-sponsored Women’s Ischemia Syndrome Evaluation (WISE). We now examine the relationship between weight cycling and prospective adverse cardiovascular outcome in this group.

**Methods:**

795 women enrolled between 1996–2001 in the WISE undergoing coronary angiography for evaluation of suspected ischemia and followed for a median of 6.0 years (interquartile range = 3.4 years). Adverse outcome was defined as a composite of all-cause death, cardiovascular mortality, non-fatal myocardial infarction, non-fatal stroke, and hospitalization for heart failure. Weight cycling was defined as the intentional loss of at least 10 lbs. (4.5 kgs.) at least three times during the women’s lifetime.

**Results:**

Women (n = 224) who reported a history of weight cycling were younger; more often white and better educated compared those without this history. At baseline, women with a weight cycling history had lower HDL-C values, higher body mass index, larger waist circumferences and higher values for fasting blood sugar, but no difference in obstructive CAD prevalence or severity. There was an inverse relationship between weight cycling and adverse **composite** cardiovascular outcome, whereby fewer of women with a history of weight cycling experienced an adverse outcome as compared to non-cyclers (21% vs 29%, respectively, p = 0.03).

**Conclusions:**

Despite an adverse association with HDL-cholesterol in women undergoing coronary angiography for suspected ischemia, weight cycling was associated with a lower adverse outcome rate in women with suspected ischemia.

## Introduction

Cardiovascular disease (CVD) remains the most common cause of death in women despite recent declines[1]. While total cholesterol and LDL-cholesterol (LDL-C) predict risk of CVD to a similar degree in women and men, fat deposition and HDL-cholesterol (HDL-C) differ between women and men, and women face a relatively higher risk associated with diabetes and elevated triglycerides, compared to men[2–7].

Prior work suggests that weight cycling, where weight is lost but regained, is associated with an elevated rate of adverse cardiovascular events[8–12], however a majority of these studies included only men. Three newer studies of weight cycling have demonstrated conflicting results; studies in middle-aged to elderly men[13] and coronary heart disease patients[14] demonstrated adverse associations with cardiovascular outcome, while a study in women failed to demonstrate any relationship[15]. Furthermore, a contemporary study of weight loss failed to demonstrate CVD benefit in subjects with diabetes[16]. Overall, these results suggest uncertainty regarding benefits/risks of weight loss and associated weight cycling for adverse cardiovascular outcomes, particularly in women. We previously reported in a cross-sectional analysis that weight cycling was associated with lower HDL-cholesterol but not the presence or severity of angiographic coronary artery disease in women with suspected ischemia undergoing coronary angiography enrolled in the NHLBI-sponsored Women’s Ischemia Syndrome Evaluation (WISE)[17]. Our current follow-up study examines follow-up prognosis to explore relationships between weight cycling and adverse cardiovascular outcome.

## Methods

The Women’s Ischemia Syndrome Evaluation (WISE) is a multi-center prospective cohort study of **935** women (enrolled from 1996–2001) designed to improve ischemic heart disease specifically in women as previously designed and described[18, 19]. The study was approved by the Cedars-Sinai Medical Center’s Institutional Review Board and all participants gave written informed consent. Inclusion criteria included women with suspected ischemia undergoing clinically indicated coronary angiography underwent an initial baseline evaluation that included demographic, medical history, risk factor, psychosocial and symptom data as well as blood samples. Body mass index (BMI) was calculated by dividing weight in kilograms by the square of height in meters; a BMI of ≥30 was defined as obese[20], while determination of waist circumference involved measurement of the waist between the umbilicus and the rib cage. Physical capacity and physical activity were measured by the Duke Activity Status Inventory and the Postmenopausal Estrogen and Progestogen Inventory questionnaires as previously described[19]. The sample for the current analyses included 795/935 (85%) enrolled WISE women with complete baseline data for weight cycling history, body mass index, HDL-C, and, angiographic evaluation.

### Definition of weight cycling

During the baseline evaluation each woman was asked about the number of times in her life that she intentionally lost a specified number of lbs. through dieting, exercise, a formal weight control program or on her own. Pregnancy and childbirth were not included. Ranges of weight were indicated rather than actual weight loss. Women were asked to select from five weight loss ranges, beginning with 10 to 19 lbs. (4.5–8.6 kgs.) and ending with 100 or more lbs. (45.5 kgs.). For the purpose of these analyses, weight cycling was defined a priori as weight loss of at least 10 lbs. (4.5 kgs.) at least 3 times[17].

### Follow-up procedures

Initial protocol-specified follow-up was conducted by experienced site nurses or physicians through direct, telephone, and/ or mail contact at 6 weeks, 1 year, and annually thereafter using a standardized scripted interview. Women were queried about symptoms, medication use, cardiovascular outcomes, hospitalizations, and diagnostic or revascularization procedures since last contact. For cases cared for a WISE clinical center, patients’ medical records were also reviewed. Median follow-up time for surviving women was 6 years (interquartile range = 3.4 years). Subsequently, we conducted a National Death Index search for all women not known to be deceased, and obtained additional death certificates. For this analysis, adverse cardiovascular outcome was defined as the first occurrence of all-cause death, cardiovascular death, nonfatal myocardial infarction, nonfatal stroke, or heart failure hospitalization.

### Statistical methods

Data are presented as means and standard deviations for continuous data and frequencies for categorical variables. Comparisons between reported weight cyclers and non-cyclers were done using the Wilcoxon two-sample test for continuous measures and the chi-square test for discrete measures. Chi square was also used in initial analyses of the association between adverse cardiovascular outcome prognosis and weight cycling. The relationship of HDL-C level to obesity and reported weight cycling was assessed using a general linear model. Standard stepwise regression methods for continuous variables were used to model HDL-C as a function of weight cycling and known modulators of HDL-C levels. The multivariable model used to evaluate weight cycling and other risk factors associated with prognosis was developed in two steps. In first step, stepwise logistic regression techniques were used to model outcome using risk factors base upon prior analyses of WISE Study data and significant univariate predictors of composite adverse events. These included obstructive CAD, age, race, education, history of diabetes, history of dyslipidemia, DASI, waist circumference, and use of aspirin. From these initial analyses, a basic model to predict outcome was created. The basic model included weight cycling, history of diabetes, DASI (functional capacity), history of dyslipidemia, education and obstructive CAD. To this basic model, baseline characteristics from Table 1 (age, race, use of alcohol, current smoking, postmenopausal status and use of hormone therapy) were added until no other variables were significant and the final model was established. The Kaplan-Meier method was used to estimate cumulative incidence rates of adverse events, with the log-rank statistic used to assess differences by history of weight cycling. All tests were two-sided and probability values ≤ 0.05 were considered statistically significant. Analyses were performed using SAS software release 9.2 and release 9.3 for windows (Cary, NC).

**Table 1:** Baseline characteristics of WISE women by history of weight cycling.

Characteristic	N	% Hx. Weigh Cycle (n = 224)	% No Hx. Weight Cycle (n = 571)	p value
**Age (years)**				
<50	196	31	69	0.04
50–64	343	31	69	
65+	256	22	78	
**Race**				
White	652	31	69	0.0002
Other	143	15	85	
**Education**				
< High School	153	20	80	0.051
High School	322	30	70	
>High School	319	30	70	
**Obese**				
BMI> = 30	322	37	63	< .0001
BMI<30	473	22	78	
**Hx. Diabetes**				
Yes	188	32	68	0.14
No	603	27	73	
**Drink Alcohol**+				
Yes	107	22	78	0.10
No	688	29	71	
**Current Smoking**				
Yes	160	25	75	0.31
No	634	29	71	
**Hx. Dyslipidemia**				
Yes	402	26	74	0.15
No	334	31	69	
**Postmenopausal**				
Yes	581	27	73	0.55
No	210	30	70	
**Use of Hormone Therapy (HT)**				
Yes	309	30	70	0.28
No	476	27	73	
Obstructive CAD*				
Yes	286	28	72	0.79
No	509	28	72	

+Defined as at least one drink per week.

*Defined as > = 50% stensosis in > = 1 coronary artery.

## Results

Among the 795 WISE included in this analysis, 224 women (28%) reported a history of weight cycling. Of these, 167 (75%) women had cycled 10–19 lbs. (4.5–8.6 kgs.), 48 (21%) had cycled 20–49 lbs. (9.1–22.3 kgs.), and 9 (4%) had cycled 50+ lbs. (22.7+ kgs.). Consistent with our prior report[17], women who reported a history of weight cycling were younger, more often white, better educated but lower HDL-C values, higher body mass index, larger waist circumferences and higher values for fasting blood sugar compared to non-cyclers. There was no group difference in the prevalence or severity of angiographic obstructive CAD (Table 1).

We stratified women into four groups, according to the presence/absence of obesity and weight cycling. The four groups were: obese/weight cycle, non-obese/weight cycle, obese/non-weight cycle and non-obese/non-weight cycle. Similar to our prior results, women who were both obese and reported a history of weight cycling had the lowest HDL-C levels, while women with neither characteristic had the highest HDL-C (Table 2). Women who cycled more weight with each cycle had lower HDL-C levels than those who cycled less weight. Women who cycled 10–20 lbs. had HDL-C levels of 52.9 ± 12.1 mg/dl, those who cycled 20–49 lbs. had levels of 51.0 ±11.1 mg/dl, those that cycled 50 lbs. or more, had HDL-C levels of 45.2 ± 6.4 mg/dl, while non-cyclers have levels of 54.7 ± 12.9 mg/dl (p<0.02).

**Table 2:** HDL-C Levels (mg/dl) by history of weight cycling and obesity status*.

Obesity (BMI ≥ 30)	Hx. Weight Cycling HDL-C (mg/dl) (n)	No Hx. Weight Cycling HDL-C (mg/dl) (n)
Yes	50.6 ± 11.0 mg/dl (n = 120)	53.3 ± 11.6 mg/dl (n = 202)
No	54.0 ± 12.4 mg/dl (n = 104)	55.4 ± 13.5 mg/dl (n = 369)

Hx. Weight Cycling

*p< 0.01

Both weight cycling and obesity were significant factors in the model.

Post hoc analyses: women who were both obese and reported weight cycling had the lowest HDL-C levels while women with neither characteristic had the highest (p = 0.0002).

Women who weight cycled more often (> 10 times) showed a trend toward lower HDL-C levels than those who cycled fewer times (6–10 times and 3–5 times) or did not cycle (51.3 ±11.3 vs. 52.2 ± 12.5 vs. 52 .7 ±11.8 vs. 54.7 ± 12.9 mg/dl, respectively, p<0.08). Table 3 depicts a stepwise regression analyses that demonstrates following adjustment for current smoking, current hormone replacement therapy, waist circumference, and alcohol use, history of weight cycling remained an independent predictor of HDL-C levels (p<0.05). Age, race, history of diabetes, obesity, and the functional status were evaluated for entry into the stepwise regression model but were not significant independent explanatory variables.

**Table 3:** Significant independent predictors of HDL-C (mg/dl) (n = 795).

Variable	Parameter Estimate (± SE)	p Value
Weight Cycling (1-yes, 0-no)	-2.0 (± 1.0)	<0.05
Current Smoking (1-yes, 0-no)	-5.6 (± 1.1)	<0.0001
Waist circumference (inches)	-0.27 (± 0.07)	<0.0001
Alcohol use* (1-yes, 0-no)	3.7 (± 1.3)	0.005
Current HRT (1-yes, 0-no)	5.3 (± 0.9)	<0.0001
Intercept	65.2 (± 2.8)	

(Linear Regression Model)

*Defined as at least 1 drink/week

HRT = hormone replacement therapy, SE-Standard Error

Age, race, history of diabetes, obese (yes/no), Duke Activity Status Inventory (a measure of functional capacity/ physical activity) were evaluated but were not significant (p<0.05) independent explanatory variables.

### Adverse outcomes and weight cycling

Among the 795 women, a total of 206 women (26%) had a combined adverse cardiovascular event during follow-up, including 141 (18%) died, 85 of whom (11%) died of cardiovascular-related causes, 28 (4%) had non-fatal MI, 39 (5%) had a non-fatal stroke, and 51 (6%) developed heart failure.

**Fig 1:**
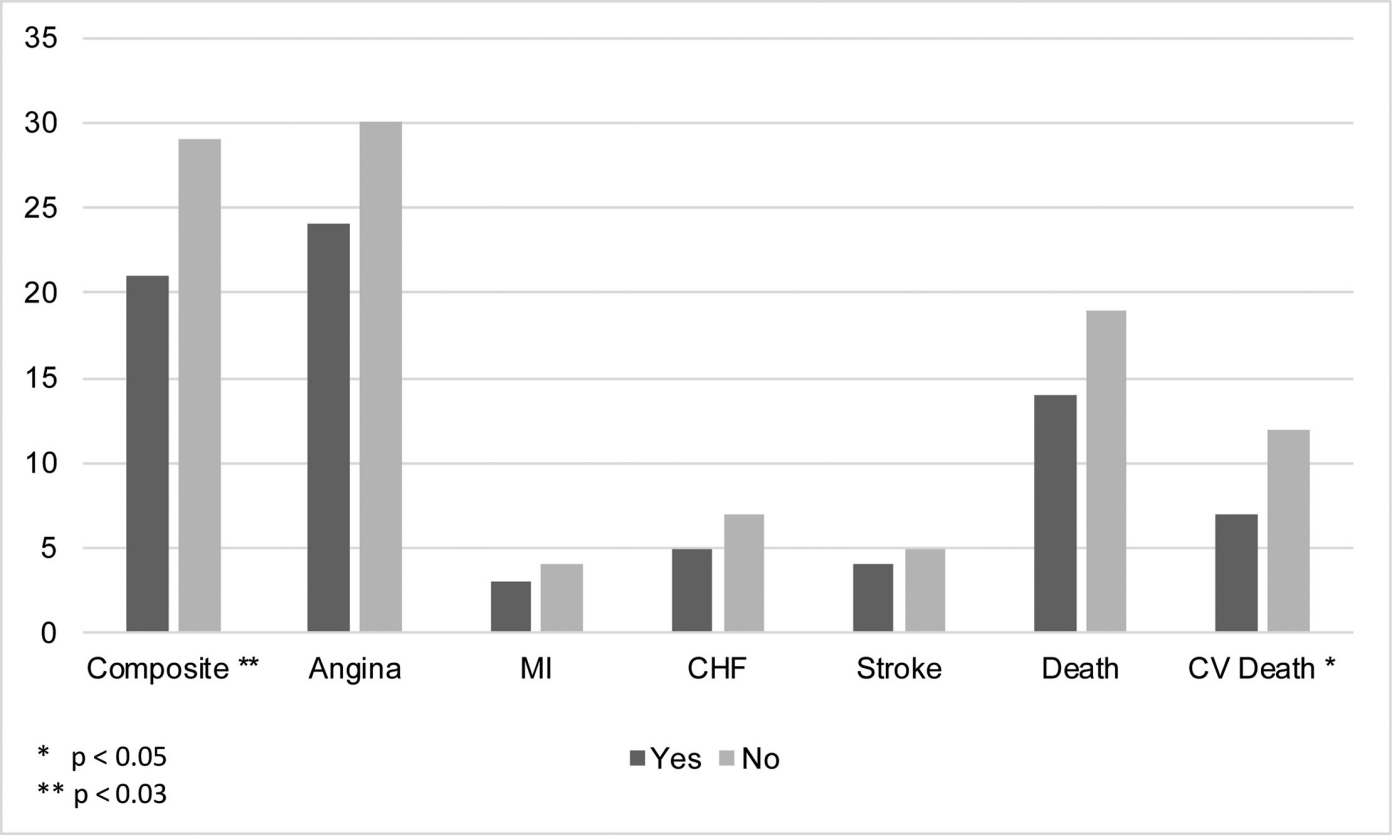
Percentage of composite cardiovascular outcomes (all cause death, non-fatal myocardial infarction, non-fatal stroke, and hospitalization for heart failure) and CV death were lower in women who reported a history of weight cycling compared to those who did not report a history of weight cycling.

**Fig 2:**
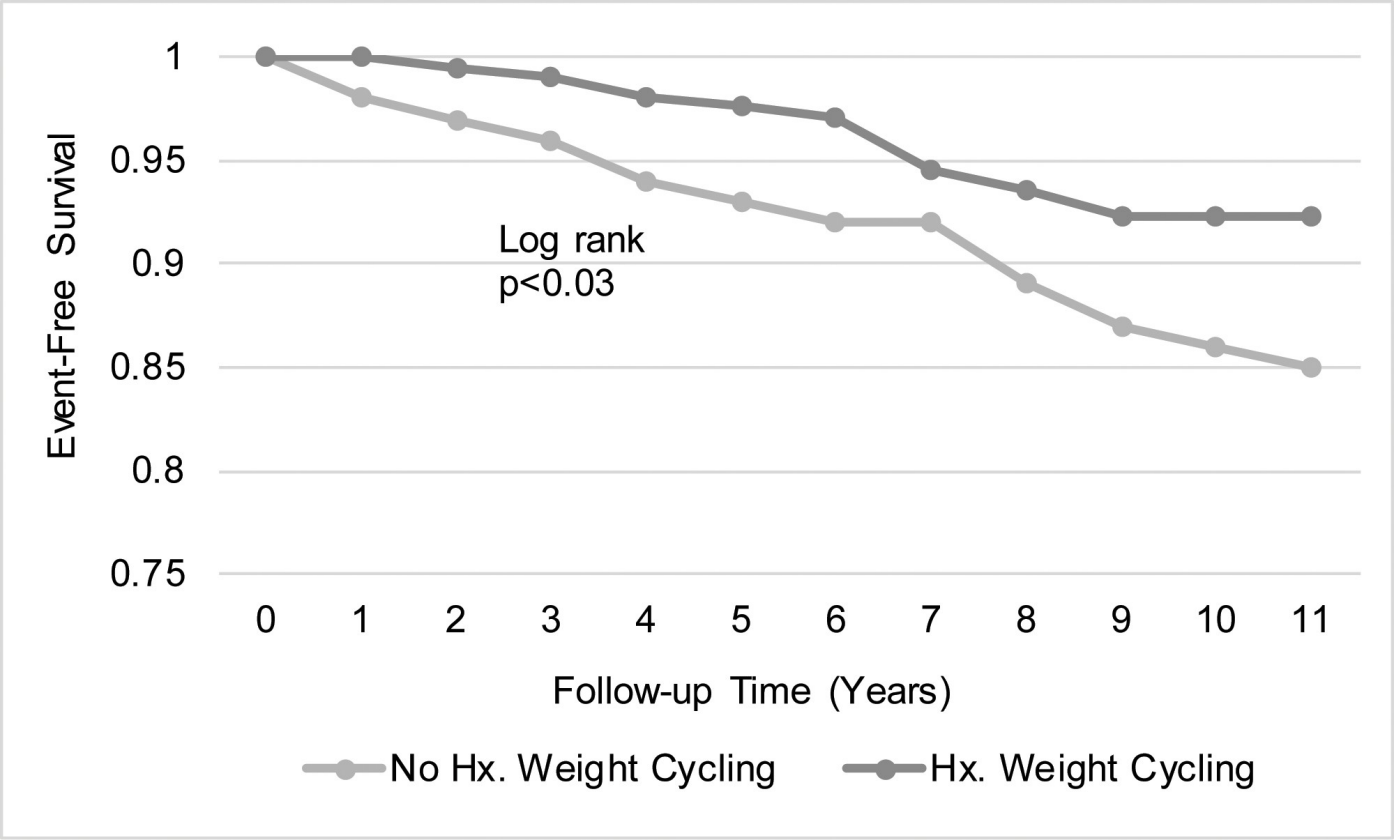
Percentage of event-free survival was higher in women who reported a history of weight cycling (n = 221) than those who did not report a history of weight cycling(n = 557). Survival curves were generated using Kaplan-Meier methods with the log rank statistic used to assess the differences.

Overall, 21% (46/221) of those with a history of weight cycling had a composite adverse cardiovascular event, compared to 29% (160/560) of those without such a history (p = 0.03). As Fig 1 indicates, similar results were found for cardiovascular deaths but not all cause mortality or other single cardiovascular outcomes. As shown in Fig 2, cardiovascular survival was better for those with a history of weight cycling vs. those without.

Because weight-cycling women differed from non-cyclers in multiple baseline characteristics (Table 4), we next evaluated the effect of age and other risk factors, in association with weight cycling and prognosis. Prior WISE analyses and other studies have shown associations of adverse cardiovascular events with age, race, history of diabetes, history of dyslipidemia, obstructive coronary artery disease (CAD), waist circumference, and DASI (functional status). Our first step in the modeling of events indicated that these variables were significantly (p<0.05) associated with the combined endpoint in univariate analyses: age (OR = 1.03, CI = 1.02–1.05), obstructive CAD (OR = 3.12, CI = 2.25–4.34), education (OR = 0.72, CI = 0.58–0.89), history of diabetes (OR = 2.97, CI = 2.09–4.23) history of dyslipidemia (OR = 2.34, CI = 1.64–3.32),DASI (functional capacity) (OR = 0.97, CI = 0.96–0.98), waist circumference (OR = 1.03, CI = 1.00–1.05), use of aspirin (OR = 1.5, CI = 1.1–2.1), and weight cycling (OR = 0.66, CI = 0.45–0.95). Based on these initial results, a basic multivariable model was established. To this basic model, baseline characteristics that could also influence outcome (Table 1) were added. As shown in Table 5, those with a history of weight cycling had a lower risk of adverse cardiovascular events that remained statistically significant even after adjustment for demographic and cardiovascular risk factors. Other analyses included two separate stepwise logistic regression models with baseline weight or baseline body mass index as possible predictors of adverse cardiovascular events. Neither baseline measure was statistically significant. Stepwise logistic regression models with cardiovascular deaths as the outcome showed weight cyclers had a lower risk of cardiovascular death (OR = 0.45, CI = 0.22–0.90) that was also statistically significant after adjustment (p<0.03).

**Table 4:** Baseline coronary risk factors by history of weight cycling.

Mean +/- SD	Hx. Weight Cycling (n = 224)	No Hx. Weight Cycling (n = 571)	P value
**Systolic BP (mmHg)**	135.6 ± 17.9	136.9 ± 21.9	0.76
**Diastolic BP (mmHg)**	77.9 ± 10.4	76.3 ± 10.9	0.05
**Fasting Blood Sugar (mg/dl)**	122.5 ± 55.6	115.2 ± 59.6	0.006
**TC (mg/dl)**	195.5 ± 44.7	195.3 ± 44.9	0.88
**Triglycerides (mg/dl)**	166.3 ± 147.2	152.8 ± 111.8	0.30
**HDL-C (mg/dl)**	52.2 ± 11.7	54.7 ± 12.9	0.03
**LDL-C (mg/dl)**	112.4 ± 40.4	111.0 ± 38.5	0.67
**Weight (lbs. and kgs.)**	182.0 ± 35.3 lbs. (82.7 ± 16.0 kgs.)	164.9 ± 36.4 lbs. (74.9 ± 16.5 kgs.)	<0.0001
**BMI**	31.8 ± 6.7	28.8 ± 6.4	<0.0001
**Waist-hip ratio**	0.84 ±.11	0.85 ± 0.10	0.14
**Waist circumference (in.)**	37.6 ± 6.7	36.2 ± 7.2	0.02
**Alcohol Intake (drinks/wk.)**	0.42 ± 2.5	0.79 ± 5.1	0.09
**Physical Activity**	7.4 ± 2.1	7.3 ± 2.0	0.32
**Functional Capacity**	20.2 ± 13.9	20.5 ± 15.2	0.76

Physical Activity assessed by the adjusted Postmenopausal Estrogen Progestogen Questionnaire; Functional Capacity assessed by the Duke Activity Status Inventory

**Table 5:** Significant independent predictors of adverse cardiovascular outcome (all-cause death, non-fatal myocardial infarction, non-fatal stroke, heart failure hospitalization).

	Odds Ratio	95% CI	p
Weight Cycling (1-yes, 0-no)	0.62	0.40–0.95	<0.03
DASI (Functional Capacity) (continuous)	0.98	0.96–0.99	<0.003
History of diabetes (1-yes, 0-no)	2.1	1.4–3.2	0.0004
Age (continuous)	1.0	1.0–1.04	<0.005
Obstructive CAD (1-yes, 0-no)	2.0	1.4–3.0	0.0004
Current smoking (1-yes 0-no)	1.9	1.2–3.0	<0.007
History of dyslipidemia	1.5	1.0–2.2	<0.05

Logistic Regression Analysis

Functional Capacity measured by Duke Activity Status Index (range: 0–58.2)

## Discussion

We report in this prognosis follow-up analysis of the NHLBI-sponsored WISE study that contrary to our hypothesis, a history of weight cycling was associated with a lower rather than higher adverse cardiovascular event rate. This lower adverse cardiovascular event rate occurred despite lower HDL-C levels and higher weight, waist circumference and blood sugar levels and was not explained by the younger age, white race, or higher education level of the weight cyclers. Specifically, significantly fewer weight cyclers experienced events compared non-weight cyclers (21% vs 29%, respectively, p = 0.03). These findings in a well-characterized cohort of women with follow-up suggest that weight cycling may not be associated with adverse health outcomes in women with risk factors and suspected ischemia.

Our current analysis of the relation between weight cycling and lower HDL-C using our complete baseline WISE sample size is consistent with our prior analysis[17]. Other prior study has also demonstrated relationships between weight cycling and cardiovascular risk factors in overweight but otherwise healthy subjects[21]. Putative mechanisms linking weight cycling to HDL-C include weight loss-associated sympathetic nervous system activation, insulin and thyroid alterations[22], and weight regain dominated by android fat deposition[23], however our current results suggest that these putative mechanistic pathways nor the observed lower level of HDL-C adversely associated with an adverse cardiovascular prognosis in women with suspected ischemia.

Our findings are not consistent with prior studies linking weight cycling with adverse cardiovascular outcomes in populations of men or predominantly men[9, 10, 12–14], but are consistent with the largest study of weight cycling which also exclusively studied women, without established CVD[15]. In 44,876 women enrolled in the Nurses’ Health Study, repeated intentional weight loss was not predictive of all-cause or cardiovascular mortality, and had a trend toward risk reduction in mild weight cyclers (RR 0.83 [CI 0.75–0.93]), similar to our current findings. Indeed, 75% (167/224) of our weight cycling women were ‘mild cyclers’ with weight loss less than 20 lbs. Notably, both our current and prior[17] results addressed “intentional” weight loss in exclusively female populations, in distinction to prior studies suggesting an adverse relationship that may have been confounded by unintentional weight loss[24–31]. These results combined with prior work in women[15] support a “not-harmful/possibly beneficial” relationship between intentional weight cycling, undertaken for weight management reasons, in women. Conversely, these results compared with prior studies in men[9, 10, 12–14], may suggest either a sex-specific adverse relationship in men, or a confounding by under-reporting of unintentional weight loss in men.

Finally, our current results combined with prior literature in women suggest that weight management that results in weight cycling does not appear to be harmful for CVD morbidity and mortality *in women*. These results further suggest that the recent clinical trial[16] of weight loss in diabetic women and men that failed to improve CVD outcomes should be carefully evaluated with regard to sex differences in weight cycling and outcomes. A totality of evidence clearly demonstrates sex differences in metabolism, fat storage, diabetes, and CVD[1–7, 32], such that further research regarding the impact of sex differences in weight reduction and weight cycling on CVD should be conducted.

## Limitations

The current study results are limited by the observational design that precludes evaluation of causality between weight cycling, and cardiovascular outcomes. This study design also limits our ability to draw conclusions regarding any pathophysiological sequencing of associations. The follow-up mean of 6 years is relatively short, and longer term follow-up analyses are always limited by survivor bias. Similarly, because WISE is a study exclusively of women and does not have a parallel group of men, findings are sex-specific and further research will be needed to understand sex differences. Also, while our findings are relevant to women undergoing coronary angiography for suspected myocardial ischemia, they may not be generalizable to more general populations of women.

## Relevance and implications

Despite an adverse association with HDL-cholesterol in women undergoing coronary angiography for suspected ischemia, weight cycling was not associated with obstructive angiographic CAD and, contrary to our hypothesis, was associated with lower rates of adverse cardiovascular outcome in WISE women with signs and symptoms of ischemia undergoing coronary angiography. Further research evaluating sex-specific relations and mechanisms between weight cycling and cardiovascular outcomes is needed.
